# Low-Crystallized Carbon as an Electron Mediator in g-C_3_N_4_/C/TiO_2_ for Enhancing Photocatalytic Degradation of Antibiotics

**DOI:** 10.3390/nano15050365

**Published:** 2025-02-27

**Authors:** Yijie Shen, Zhe Liu, Renke Bi, Bianbian Zhou, Yan Wang, Jialong Liu, Zhiyu Wang, Bing Han

**Affiliations:** 1 State Key Laboratory of Silicon and Advanced Semiconductor Materials, School of Materials Science and Engineering, Zhejiang University, Hangzhou 310027, China; 3190100542@zju.edu.cn (Y.S.); 21926082@zju.edu.cn (Z.L.);; 2 College of Sciences, College of Forestry, Henan Agricultural University, Zhengzhou 450002, China

**Keywords:** photocatalytic, g-C_3_N_4_/C/TiO_2_, low-crystallized carbon, electron mediator, tetracycline hydrochloride

## Abstract

Photodegradation of antibiotics based on photocatalytic semiconductors is a promising option to alleviate water pollution. Despite its limitations, TiO_2_-based photocatalysts are still the most widely studied materials for pollutant degradation. In this work, a pomegranate-like g-C_3_N_4_/C/TiO_2_ nano-heterojunction was constructed using the hydrothermal–calcination method, consisting of interconnected small crystals with a dense structure and closely contacted interface. Low-crystallized carbon filled the gap between TiO_2_ and g-C_3_N_4_, forming a large interface. The local in-plane heterostructures generated by C/g-C_3_N_4_ are further improved for carrier transport. As expected, the optimal sample calcined at 300 °C (GTC-300) efficiently eliminated tetracycline hydrochloride (TC-HCl, 20 mg L^−1^), achieving a removal rate of up to 92.9% within 40 min under full-spectrum irradiation and 87.8% within 60 min under the visible spectrum (λ > 400 nm). The electron mediator, low-crystallized carbon, successfully promoted the formation of new internal electric fields via the widespread heterojunction interface, which accelerated the separation and migration of photogenerated carriers between g-C_3_N_4_ and TiO_2_. These results confirm that the g-C_3_N_4_/C/TiO_2_ nano-heterojunction exhibited outstanding photodegradation performance of TC-HCl. The electron mediator shows great potential in promoting carrier transfer and enhancing photocatalytic performance of heterogeneous photocatalysts in water treatment.

## 1. Introduction

Freshwater resources are currently in short supply, with only 0.5% of the world’s water available for human consumption [1]. Even at extremely low concentrations, the presence of medicines in the aquatic environment offers significant difficulties, including chronic toxicity, endocrine disruption, and the development of disease resistance [2,3,4]. Tetracycline hydrochloride (TC-HCl) is a commonly used antibiotic with bacteriostatic qualities, but it has emerged as a new and dangerous pollutant due to its overuse and difficulty in degradation [5,6,7,8,9]. In the pursuit of an efficient, cost-effective, and environmentally friendly solution, photocatalysis based on semiconductors has been highlighted as a potential choice when compared to existing approaches such as physical adsorption and biological degradation [10,11].

TiO_2_ is one of the most typical and investigated photocatalysts in water pollutant degradation due to its unique advantages under ultraviolet light irradiation [3,12,13]. Meanwhile, its broad bandgap (3.2 eV) significantly limits its ability to harness visible light from the solar spectrum. To address this issue, a variety of narrow-bandgap semiconductors were used to create an extensive amount of TiO_2_-based photocatalysts, which can be classified as various types of heterojunctions such as type-II, p-n, Schottky, and Z-scheme. g-C_3_N_4_ is a metal-free polymeric semiconductor, exhibiting appealing electronic band arrangement and a moderate band gap of 2.7–2.8 eV corresponding to visible light [14,15,16,17]. Synergistic interactions between TiO_2_ and g-C_3_N_4_ can be leveraged to combine their redox capacities through the building of an artificial Z-scheme heterojunction. The visible light response Z-scheme heterojunction TiO_2_/g-C_3_N_4_ has been successfully created by coupling the matching energy band of g-C_3_N_4_ and TiO_2_, yielding superior photocatalytic performance in comparison to TiO_2_ [18,19,20,21].

Numerous research papers have explored the design and synthesis of TiO_2_/g-C_3_N_4_ Z-scheme heterojunction photocatalysts, which can be broadly categorized into three general strategies based on the sequence of component preparation: physical mixing of separately prepared g-C_3_N_4_ and TiO_2_ [22], in situ growth of TiO_2_ on pre-existing g-C_3_N_4_ [23], and loading g-C_3_N_4_ onto TiO_2_ [24]. The primary objective of each of these methods is to create a heterojunction by bringing TiO_2_ and g-C_3_N_4_ into contact with each other. Despite the fact that the heterojunction is capable of charge separation, the interface between TiO_2_ and g-C_3_N_4_ restricts the total charge separation efficiency. As a result, all-solid-state Z-scheme heterojunction is commonly used to ensure photocatalytic function in these photocatalysts.

As an electrical mediator, noble metals, such as Ag or Au nanoparticles, are commonly used to bridge two semiconductors [25,26] to promote efficient Z-scheme charge separation and migration at the heterointerface, enhancing photocatalytic performance. But this must contend with issues of high cost and limited availability, which restricts their use in photodegradation [27]. Researchers have thus sought to replace them with less expensive materials that have comparable electron transport characteristics. Amorphous or low-crystallized carbon is a low-cost and widely available carbon material that allows for charge carrier separation and transfer [13,28,29]; for example, it possesses a hexagonal molecular shape identical to g-C_3_N_4_ and numerous sp^2^-hybridized π conjugation bonds [28]. As consequence, this experiment intends to use low-crystallized carbon as the electrical mediator in an all-solid-state Z-scheme heterojunction rather than noble metals to improve photocatalytic performance.

Based on our previous experience and continuing interest in this field [30], g-C_3_N_4_/C/TiO_2_ all-solid-state Z-scheme heterojunction was designed in situ and synthesized via a hydrothermal–calcination reaction. The as-synthesized g-C_3_N_4_/C/TiO_2_ Z-scheme heterojunction is firmly constructed by interconnected small crystals, presenting a typical pomegranate shape, which broadens the excitation wavelength range, facilitates surface reaction sites, and increases redox capacity. Low-crystallized carbon filled the gap between TiO_2_ grains and g-C_3_N_4_, serving as a solid-state electron mediator in the Z-scheme structure to enhance photocarrier separation efficiency. TC-HCl was used as an organic pollutant to explore the photocatalytic degradation performance. Particularly, the g-C_3_N_4_/C/TiO_2_ holds great potential for the development of high-performance heterogeneous photocatalysts and large-scale sustainable application of water pollutant photodegradation.

## 2. Materials and Methods

### 2.1. Materials

Titanium isopropoxide (TTIP, 95%), 2-cyanoguanidine (DCD 99%), and tetramethylammonium hydroxide (TMAH, 10% aqueous solution) were purchased from Shanghai Aladdin BioChem Technology Co., Ltd. (Shanghai, China). Urea (AR, ≥99.0%), ethylene glycol (EG, AR, ≥99.5%), isopropanol (AR, ≥99.7%), ethanol (CP, 99.8%), ethylenediaminetetraacetic acid disodium (Na-EDTA, AR, 99%), p-benzoquinone (p-BQ, AR, ≥98.0%), isopropyl alcohol (IPA, AR, ≥99.7%), and silver nitrate (AgNO3, AR, 99.8%) were obtained from Sinopharm Chemical Reagents Co., Ltd. (Beijing, China). All reagents were not further purified before use, and deionized water (DI) was used throughout the experiment.

### 2.2. Methods

#### 2.2.1. Synthesis

Initially, 10 mL of TMAH was mixed with 30 mL of EG and sonicated uniformly; this solution was designated as solution A. After adding 5 g of DCD, 2 mL of TTIP was gradually added to the former solution while stirring continuously. The mixed solution (solution B) was placed in a polytetrafluoroethylene hydrothermal reactor and heated to 200 °C for 8 h. After the reaction, the product was washed with DI and 100% ethanal and dried at 80 °C to produce the intermediate. The intermediate was subjected to various thermal condensation temperatures (300 and 500 °C) for 2 h under ambient air conditions in a muffle furnace, producing powder samples called GTC-300 and GTC-500.

For comparison, the synthesis of TiO_2_/C powders (sample without g-C_3_N_4_) followed a different procedure [31]: 2 mL of TTIP was slowly added to solution A and stirred until clarity was achieved. Subsequently, the hydrothermal reaction and thermal polymerization were conducted under the same conditions as previously described. g-C_3_N_4_ was synthesized by heating 10 g of urea in a muffle furnace at 550 °C for 2 h. The resulting product was cooled to room temperature and subsequently ground into a fine powder.

#### 2.2.2. Characterization

X-ray diffraction (XRD) analysis was conducted using a Shimadzu XRD-7000 X-ray diffractometer, equipped with a Cu-Kα radiation source (λ = 1.5406 Å), within a 2θ range of 10° to 80°. Scanning electron microscopy (SEM) images were captured with a Zeiss Sigma 300 scanning electron microscope. Transmission electron microscopy (TEM) imaging and elemental analysis were carried out with a JEM-2100 transmission electron microscope. X-ray photoelectron spectroscopy (XPS) spectra were obtained using a Thermo Scientific Thermo K-Alpha (Waltham, MA, USA), with binding energies referenced to the C 1s peak at 284.8 eV. UV–Vis and UV–Vis diffuse reflectance (UV–Vis/DRS) spectra were recorded on a BeiFen Ruili UV-1601 spectrophotometer, with wavelength ranges of 200–800 nm and 250–700 nm, respectively [30]. Photoluminescence (PL) spectra were measured using a Hitachi F-4600 fluorescence spectrometer (Tokyo, Japan), using a Xe lamp (excitation at 365 nm) as a light source. The specific surface area of the nanocomposite was determined through N_2_ adsorption at 77 K, using a Brunauer–Emmett–Teller (BET) analyzer (Micromeritics ASAP 2460, Norcross, GA, USA). Degassing was performed at 200 °C for 8 h before measurement. Raman spectra were collected with a Horiba LabRAM HR Evolution (Kyoto, Japan) using a 532 nm detection laser. Thermogravimetric analysis (TGA) was performed with a Netzsch TGA209F1 (Selb, Germany), ranging from 300 °C to 800 °C at a constant heating rate of 10 °C min^−1^ in air. The work functions were measured using ultraviolet photoelectron spectroscopy (UPS, Thermo Fisher Scientific ESCALAB Xi, Waltham, MA, USA) with a He I source (excitation energy of 21.2 eV) with a polarization potential (bias) of −5.0 eV.

#### 2.2.3. Photocatalytic Performance and Active Species Measurements

The photocatalytic performance was assessed by degrading the pollutants TC-HCl (20 mg L^−1^) and methylene blue (MB, 20 mg L^−1^) in a solution with 20 mg of photocatalyst. Prior to irradiation, the mixed solution was darkened and vigorously stirred for 40 min to achieve adsorption–desorption equilibrium. The mixed solution was subjected to a 300 W Xe lamp (PLS-SXE 300, Beijing, China), with no additional filter for UV–visible light experiments and an additional wavelength λ > 400 nm optical filter for visible light experiments. At regular intervals, a 3 mL sample of the solution was removed and centrifuged to separate the photocatalyst. The concentration of contaminants in the solution was then determined using UV–Vis spectrophotometry (BeiFen Ruili UV-1601, Beijing, China).

To identify the critical active species, multiple controlled experiments were performed. IPA, BQ, AgNO_3_, and EDTA-2Na were introduced as scavengers of ·OH (hydroxyl radical), ·O^2−^ (superoxide radical), h^+^ (hole), and e^−^ (electron), respectively. Radicals (·O^2−^ and ·OH) were investigated using electronic spin resonance (ESR, Bruker EMXplus-6/1), in which the ·O^2−^ and ·OH can be captured by 5,5-dimethyl-1-pyrroline 1-oxide (DMPO).

## 3. Results and Discussion

**Figure 1:**
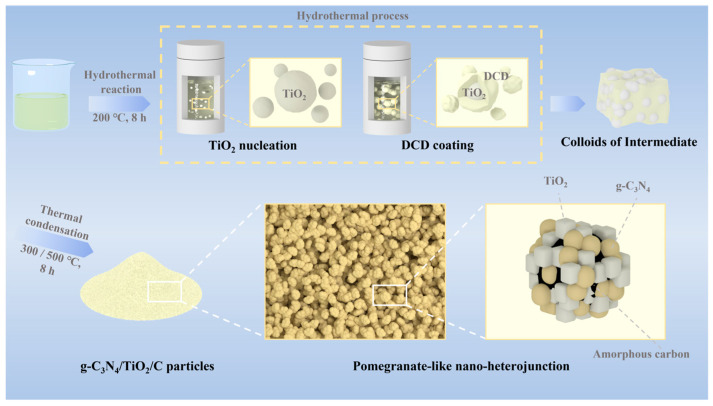
Schematic illustration of g-C_3_N_4_/C/TiO_2_ prepared using a hydrothermal process.

The schematic diagram in Figure 1 illustrates the hydrothermal–calcination synthesis method. During the hydrothermal process, nano-TiO_2_ nucleates with a diameter of 5–10 nm. Following that, TMAH as a surfactant allows nitrogen source (DCD) adsorption on its surface. After DCD coating, the dispersed particles become complex and then colloid, resulting in an intermediate powder. The subsequent heat treatment caused TMAH and DCD to undergo simultaneous thermal condensation, resulting in the formation of carbon and g-C_3_N_4_, as well as promoting the crystal growth and recombination of nano-TiO_2_. This resulted in a pomegranate-like structure composed of well-dispersed g-C_3_N_4_, TiO_2_, and carbon.

### 3.1. Structure and Morphology Characteristics

**Figure 2:**
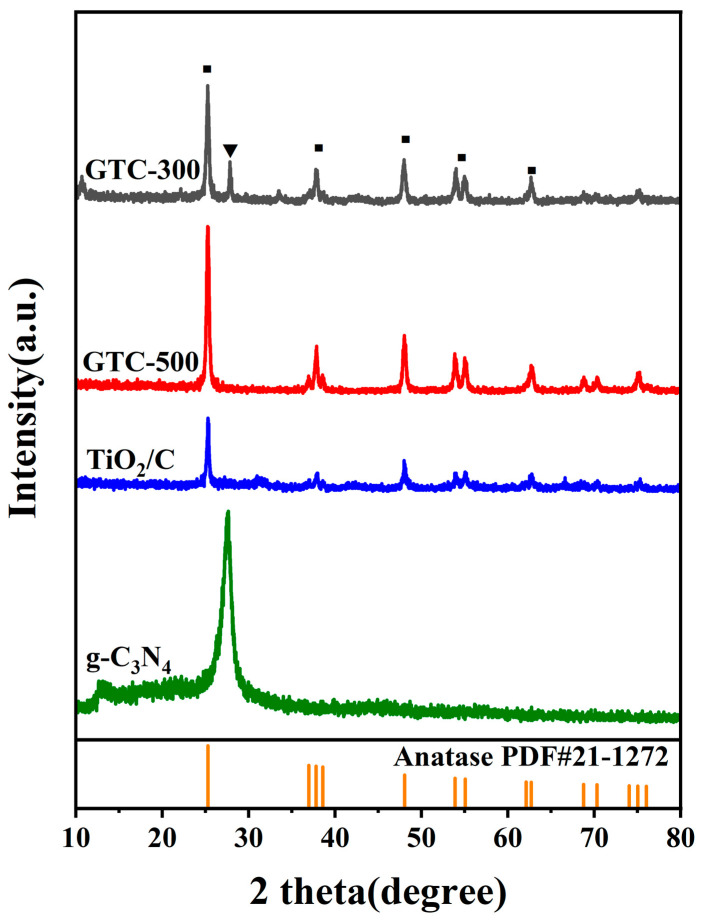
XRD patterns of GTC-300, GTC-500 TiO_2_/C, and g-C_3_N_4_ (anatase phase TiO_2_ marked with ■, g-C_3_N_4_ marked with ▼, amorphous carbon marked with *).

A range of tests are used to cross-verify both the composition and the structure of GTC-300. Firstly, the corresponding XRD patterns of GTC-300, GTC-500, TiO_2_/C, and g-C_3_N_4_ are shown in Figure 2. It is found that GTC-300, GTC-500, and TiO_2_/C all contain anatase phase TiO_2_ compared with the standard anatase TiO_2_ card (JCPDS.21-1272). Another diffraction peak of GTC-300 at 27.3 was found belonging to the (002) crystal plane of g-C_3_N_4_ (JCPDS.87-1526). This peak arises from the periodic stacking of conjugated aromatic structures along the c-axis [32], indicating its characteristic graphitic layer structure. Through thermogravimetric experiments, we speculate that the TiO_2_ content in GTC-300 is about 35%, the g-C_3_N_4_ content is about 20% (550 °C to 700 °C), and the carbon content is about 40% (300 °C to 450 °C), while GTC-500 is about 99% TiO_2_ (Figure S1). These results confirm the absence of g-C_3_N_4_ in the GTC-500 sample. Due to the small size and close interface contact of the intermediates, a lower g-C_3_N_4_ synthesis temperature is required compared to traditional formation [15].

**Figure 3:**
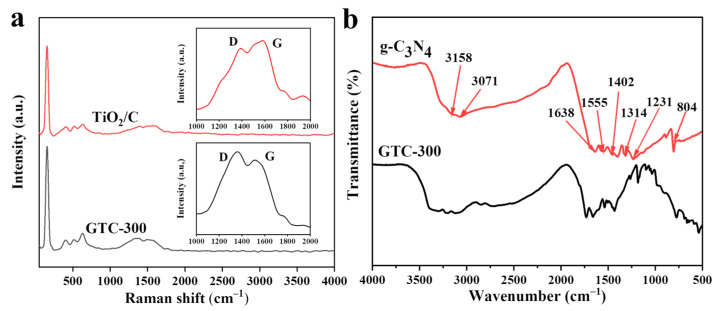
(**a**) Raman spectra of GTC-300 and TiO_2_/C and Raman shift of 1000–2000 cm^−1^ enlarged image. (**b**) FT-IR pattern of GTC-300 and g-C_3_N_4_ nanoparticles.

To obtain detailed information about carbon and further investigate the local structure of GTC-300 and TiO_2_/C, Raman characterization was performed (Figure 3a). The typical characteristic peaks of anatase at 144, 399, 515, and 639 cm^−1^ could be observed in both the GTC-300 and TiO_2_/C spectra [33,34]. Two prominent reflections at 1365 cm^−1^ and 1596 cm^−1^ in the GTC-300 and TiO_2_/C spectra are assigned to the D (disordered carbon) and G (graphitized carbon) bands of the carbon coating. The ID/IG intensity ratio of 3.59 calculated from the GTC-300 spectrum confirms the presence of low-crystallized carbon. Thus, the structure of GTC-300 has been successfully established by g-C_3_N_4_, TiO_2_, and low-crystallized carbon.

GTC-300 was further characterized by FT-IR spectra that show no obvious change compared to g-C_3_N_4_ (Figure 3b). For g-C_3_N_4_, the strong band near 810 cm^−1^ represents the typical vibration mode of the triazine structure [35]. The bands at 1638 cm^−1^, 1555 cm^−1^, and 1402 cm^−1^ belong to the skeletal vibrations (aromatic C-N). The bands at 1314 cm^−1^ and 1231 cm^−1^ are attributed to the stretching vibration of the connecting units of fully condensed N-(C)_3_ and partially condensed C-N-H [36]. The absorbance in the range of 3000~3400 cm^−1^ is related to the residual N-H groups and O-H bands [37]. Comparison shows that the FT-IR curve of GTC-300 exhibits no obvious difference from that of g-C_3_N_4_, indicating that GTC-300 contains a composite structure of g-C_3_N_4_ and TiO_2_ with the skeleton structure of g-C_3_N_4_.

**Figure 4:**
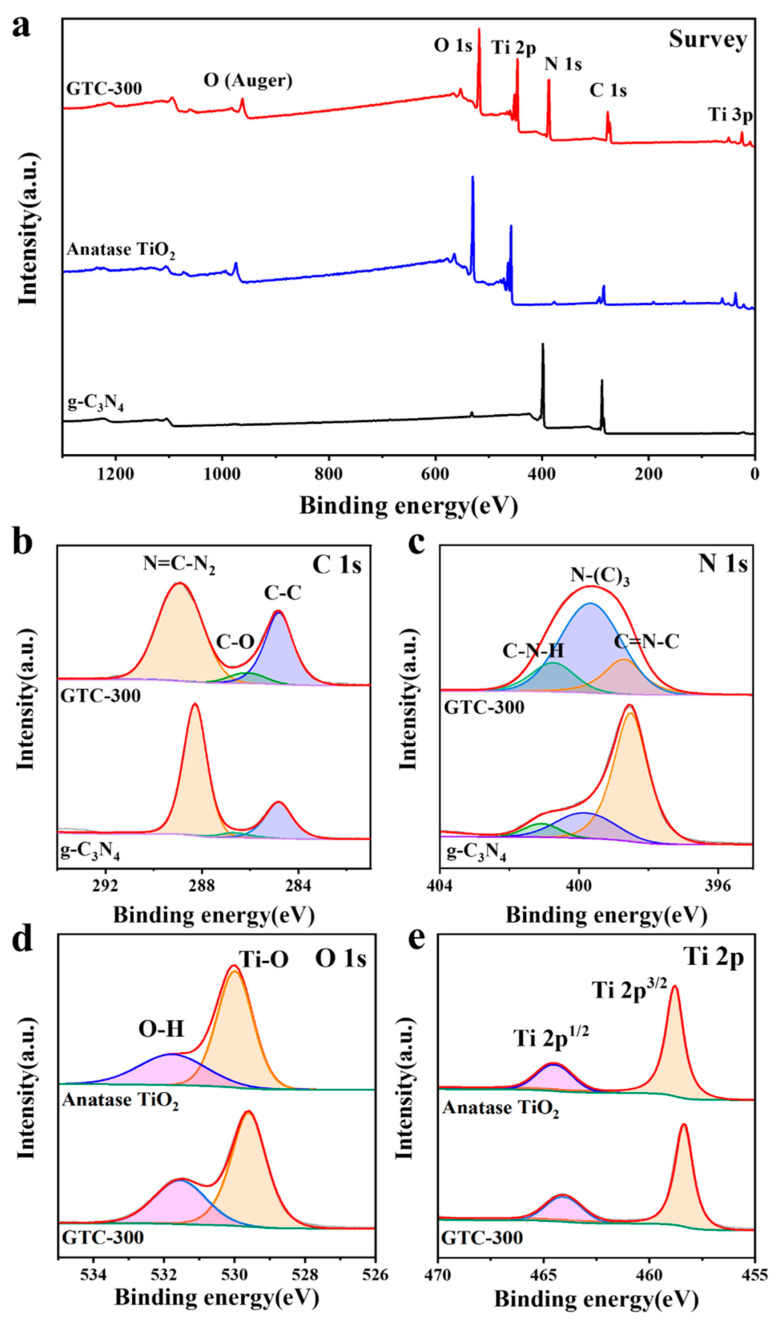
XPS of (**a**) survey, (**b**) C 1s, (**c**) N 1s, (**d**) O 1s, and (**e**) Ti 2p for GTC-300, g-C_3_N_4_, and anatase TiO_2_ nanoparticles.

The XPS spectra were measured to analyze the chemical states of different elements of the samples. In the full survey of GTC-300 (Figure 4a), signals of C, N, O, and Ti were investigated. For the C 1s spectrum in Figure 4b, the obvious peaks of both GTC-300 and g-C_3_N_4_ at 284.8 eV, 286.1 eV, and 288.6 eV could be attributed to C-C or adventitious C contamination, C-O, and N=C-N2 bonds, respectively [37,38,39]. The relative intensity of the binding energy peak at 284.8 eV and 286.1 eV in GTC-300 increases to that of g-C_3_N_4_, indicating the higher proportion of C-C or adventitious C contamination and C-O. Combined with Raman results, low-crystallized carbon is considered to be introduced, reducing the relative ratio of N=C-N_2_ bonds. The N 1s spectrum reveals three peaks at 398.7 eV, 399.7 eV, and 401.1 eV (Figure 4c), representing sp^2^ hybridized C=N-C structure, N-(C)_3_ group, and C-N-H structure, respectively [38,39]. Among them, the binding energy peak of 399.7 eV is significantly higher than the other two, indicating that low-crystallized carbon tends to be inserted at the C=N-C and C-N-H structures. Its incorporation destroys the long-range structure of g-C_3_N_4_, causing the proportion of N-(C)_3_ groups to increase significantly. In Figure 4d, the O 1s spectrum exhibits peaks at 529.8 eV and 531.8 eV, which are associated with the Ti-O bonds within TiO_2_ and the hydroxyl groups or water molecules present on the surface. Compared with the concentrated absorption process [39], the significantly higher peak intensity at 531.8 eV is attributed to the incorporated low-crystallized carbon structure, predicting a porous surface with increased water and hydroxyl group absorption. The Ti 2p spectrum consists of binding energy peaks at 458.5 eV and 464.2 eV (Figure 4e), corresponding to Ti 2p_3/2_ and Ti 2p_1/2_ valence states, respectively. The results obtained from XPS analysis suggest the desirable interrelation between TiO_2_, g-C_3_N_4_, and low-crystallized carbon.

In addition, the specific surface area of g-C_3_N_4_/C/TiO_2_ nano-heterojunction is calculated by the Brunauer–Emmett–Teller (BET) equation through N_2_ adsorption–desorption isotherm measurement. As displayed in Figure S2, the surface area of TiO_2_/C and P25 is 126.41 m^2^/g and 30.21 m^2^/g, respectively. GTC-300 has a specific surface area of 31.86 m^2^/g, due to its dense structure and closely contacted interface. This feature sacrifices surface area but facilitates interfacial carrier transport, thus facilitating photocatalysis.

**Figure 5:**
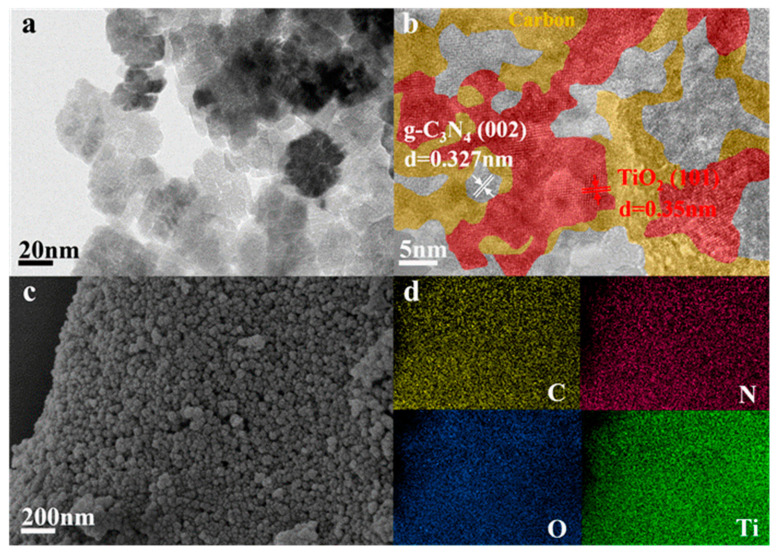
(**a**) TEM, (**b**) HRTEM, (**c**) SEM, and (**d**) EDS of GTC-300 nanoparticles.

To provide direct evidence for successful construction of the g-C_3_N_4_/C/TiO_2_ nano-heterojunction, SEM, TEM, and HRTEM characterization was conducted. As shown in Figure 5a, GTC-300 presents a typical pomegranate-like structure, a composition of interconnected small crystals, ranging from 20 to 40 nanometers in diameter and held together by specific substances. Further observation by HRTEM (Figure 5b) reveals a clear lattice distance of 0.353 and 0.327 nm, corresponding to the (101) plane of anatase TiO_2_ and the (002) plane of g-C_3_N_4_. The size of TiO_2_ is only 5–10 nanometers; it is speculated that DCD coating inhibits its further growth. Simultaneously, numerous substances exhibiting low-crystallized fringes are scattered amidst TiO_2_ and g-C_3_N_4_. According to the above results, these low-crystallized substances are considered to be low-crystallized carbon, which is deposited on the surface of anatase grains and g-C_3_N_4_ or filled between them, constructing the nanocomposite structure of g-C_3_N_4_, anatase TiO_2_, and carbon. The interface structure formed between the g-C_3_N_4_ lattice, TiO_2_ lattice, and lattice could be clearly observed, and different components are in close contact. Moreover, low-crystallized carbon and g-C_3_N_4_ formed distinct local in-plane heterostructures. The SEM image in Figure 5c exhibits near-spherical cubes tightly united by many small spheres, resulting in a rough surface with gaps, confirming the pomegranate-like structure observed using TEM. Moreover, the EDS mapping image indicated the four main elements (C, N, O, and Ti) were uniformly dispersed in the GTC-300 sample (Figure 5d). Thus, the above characterization results altogether demonstrate that the g-C_3_N_4_/C/TiO_2_ nano-heterojunction has been successfully constructed by hydrothermal–calcination synthesis.

### 3.2. Optical Properties

**Figure 6:**
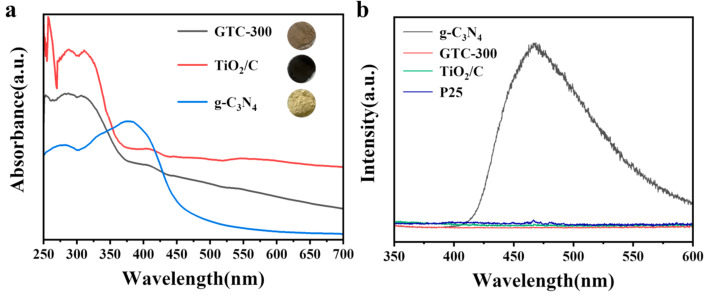
(**a**) UV-DRS spectra and (**b**) photoluminescence (PL) spectra for GTC-300, TiO_2_/C, g-C_3_N_4_, and P25 nanoparticles.

As shown in the UV–Vis absorption spectra of GTC-300, TiO_2_/C, and g-C_3_N_4_ powder in Figure 6a, g-C_3_N_4_ absorbs light from the visible region while GTC-300 and TiO_2_/C exhibit absorption across the entire spectrum within 250 to 700 nm, attributed to the presence of low-crystallized carbon with excellent light-absorbing properties. Combining the color of optical photos of powder samples, GTC-300 is brown, TiO_2_/C is black, and g-C_3_N_4_ is yellow; the light absorption capacity of GTC-300 falls in between that of TiO_2_/C and g-C_3_N_4_ and aligns with the results of the UV–Vis absorption spectra.

According to the curves of (αhν)^1/2^ and hν converted using the Kubelka–Munk function [40], as shown in Figure S3, GTC-300 showed a smaller Eg value of approximately 2.3 eV than the commercial TiO_2_ (3.2 eV), which has a relatively narrower band gap. It was anticipated that GTC-300’s increased absorption efficiency of visible light would accelerate the degradation of organic pollutants.

The PL spectra were measured to detect the charge recombination behaviors and lifetime of photogenerated charge in samples (Figure 6b). g-C_3_N_4_ has a strong emission peak near 460 nm, due to the recombination of photogenerated electron-holes inside g-C_3_N_4_, while GTC-300, TiO_2_/C, and P25 all exhibit minimal emission PL peaks at 460 nm. In the enlarged PL spectrum (Figure S4), it can be found that the emission peak intensity of the g-C_3_N_4_/C/TiO_2_ nano-heterojunction is lower than that of P25. Among the samples, it is worth noting that GTC-300 produces the weakest PL signal at almost any wavelength, indicating the least recombination of photogenerated charge and the most robust capacity for the separation and migration of charge. This implies that the heterojunction structure and the robust conductivity of carbon significantly enhance the migration of photogenerated carriers within the material while spatially separating these carriers, ultimately bestowing the material with exceptional photocatalytic performance.

### 3.3. Photocatalytic Performances

To investigate the photocatalytic performance of photocatalysts synthesized via the hydrothermal–calcination method, degradation experiments have been conducted targeting water contaminants TC-HCl and MB under simulated sunlight and visible light, respectively. The mixed solution underwent a dark treatment with vigorous stirring for 40 min to reach an adsorption–desorption equilibrium state before irradiation.

**Figure 7:**
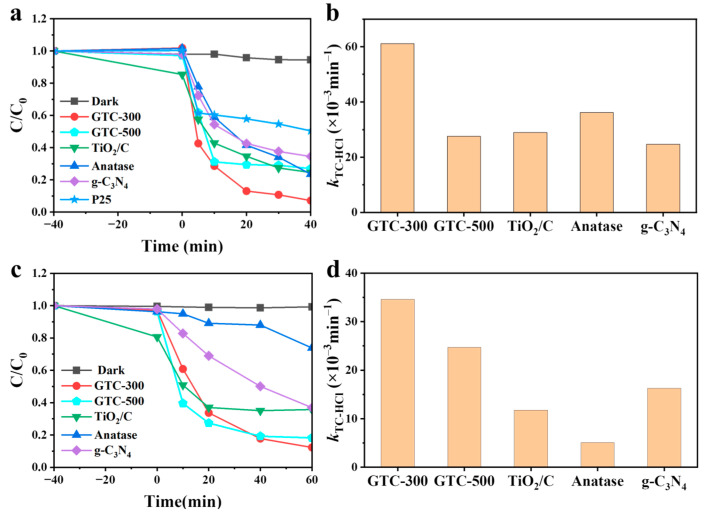
Photocatalytic degradation TC-HCl curves under simulated sunlight irradiation (**a**) and visible light irradiation (**c**) and reaction rate constant for degradation TC-HCl under simulated sunlight irradiation (**b**) and visible light irradiation (**d**) over GTC-300, GTC-500, TiO_2_/C, anatase, and g-C_3_N_4_ nanoparticles.

The original UV–Vis spectra used to monitor the TC-HCl concentration in the solution are displayed in Figure S5; the two main absorption peaks of TC-HCl exhibit a declining pattern as the illumination time was extended, suggesting that the samples effectively facilitated the complete degradation of TC-HCl. The concentration versus time curve under simulated sunlight is presented in Figure 7a. As can be observed, TC-HCl hardly decomposed, and the adsorption of TC-HCl on GTC -300 was also negligible without irradiation. TiO_2_/C, g-C_3_N_4_, and GTC-500 all presented limited degradation capacity, with degradation rates of 75.4%, 65.5%, and 73.0%, respectively. Conversely, GTC-300 presented efficient degradation, achieving a rate of 92.9% under the same condition. Another set of data under visible irradiation exhibited a similar pattern (Figure 7c). After 60 min of degradation, TiO_2_/C and g-C_3_N_4_ only degraded 64.3% and 63.1% of TC-HCl, while GTC-300 and GTC-500 degraded 87.7% and 81.8%, indicating that GTC-300 did possess the best degradation capacity over all samples in both sunlight and visible light conditions.

Based on pseudo-first-order kinetics, the degradation procedure has been further described to calculate the reaction rate constant (*k*_TC-HCl_) shown in Figure 7b,d. Among the samples, GTC-300 displays the best apparent rate constant *k*_TC-HCl_ with 61.1 × 10^−3^ min^−1^ and 34.6 × 10^−3^ min^−1^ under UV–visible light and visible light irradiation, which is 2.1 and 2.5 times higher than TiO_2_/C (28.9 × 10^−3^ min^−1^) and g-C_3_N_4_ (24.7 × 10^−3^ min^−1^) under UV–visible light irradiation and 2.9 and 2.1 times higher than TiO_2_/C (11.8 × 10^−3^ min^−1^) and g-C_3_N_4_ (16.3 × 10^−3^ min^−1^) under visible light irradiation. The results of gradation efficiency and kinetics are consistent with the characterization results, verifying that the g-C_3_N_4_/C/TiO_2_ nano-heterojunction successfully broadens the excitation wavelength range and improves the efficiency of charge separation to achieve an excellent photocatalytic performance. After five cycles of testing, the degradation rate can still reach 88.9% (Figure S6a). After the samples were collected by centrifugation after photocatalysis, the XRD results showed that there are no obvious peaks changed, indicating that GTC-300 has high stability (Figure S6b).

Comparing this study to others, Table 1 lists the earlier research findings on the degradation of TC-HCl when TiO_2_ is combined with other metals or semiconductors. Clearly, our work’s TC-HCl degradation process takes less time than previous studies that use visible light irradiation, and it also degrades at a faster rate for the same amount of time (40 min).

Moreover, we selected dyes as additional pollutants to help demonstrate the photocatalytic performance. As a target of MB, the samples synthesized via the hydrothermal–calcination method exhibit strong catalytic ability. With UV–visible light irradiation, MB had been almost completely degraded in 20 min with GTC-300 (Figure S7). Furthermore, the apparent rate constant *k*_MB_ of GTC-300 reaches 154.6 × 10^−3^ min^−1^, which is 3.3 times that of TiO_2_/C and 5.1 times that of g-C_3_N_4_ (Figure S7d), indicating a significant contrast. The photocatalytic degradation performance of GTC-300 is significantly better than that of TiO_2_/C from our previous work [31].

### 3.4. Reaction Mechanisms

**Figure 8:**
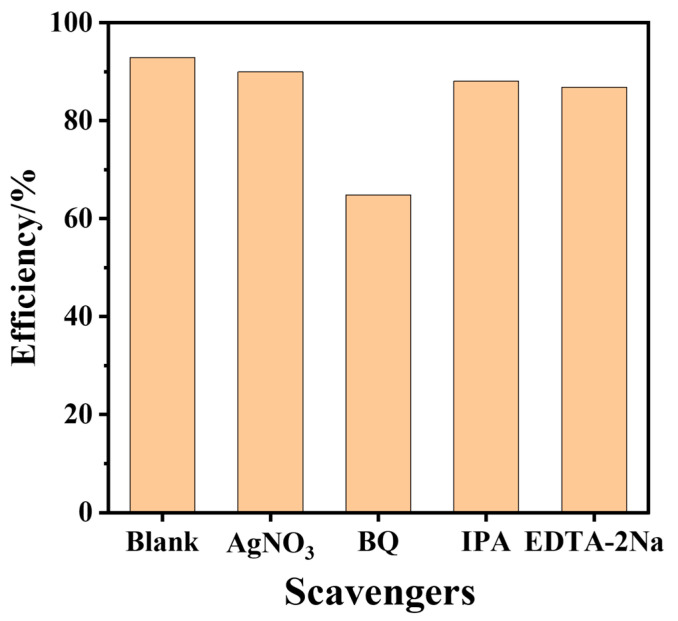
The active species capture experiment of TC-HCl photodegradation by GTC-300.

**Figure 9:**
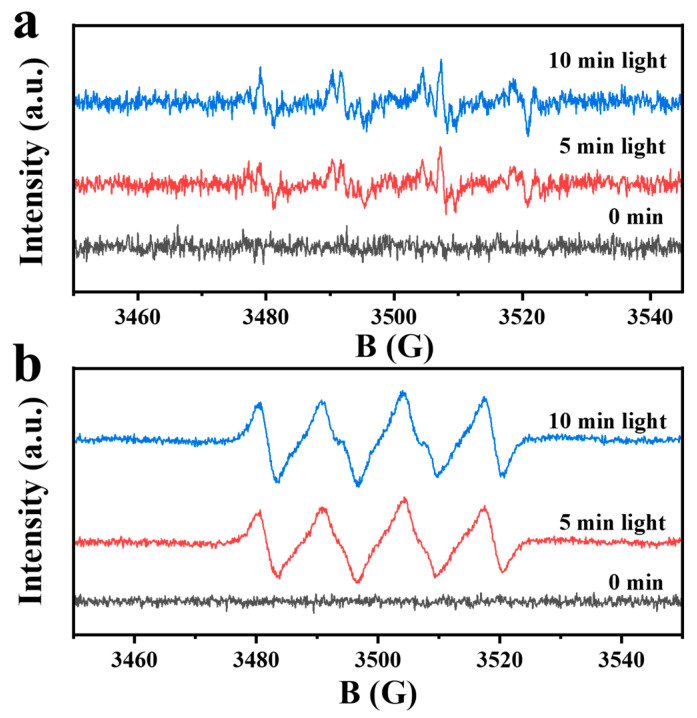
DMPO spin-trapping ESR spectra of GTC-300 in (**a**) aqueous dispersion for DMPO–·OH and (**b**) methanol dispersion for DMPO–·O_2_^−^.

To determine the dominant active substance in the photocatalytic process, an active species capture was conducted, explaining the mechanism of the photolytic reaction. TC-HCl is used as the degraded pollutant, in which AgNO_3_, Na-EDTA, IPA, and p-BQ were used as radical scavengers for holes (h^+^), electron (e^−^), hydroxide (·OH), and superoxide radicals (·O_2_^−^) species, respectively. As shown in Figure 8, the degradation performance was slightly decreased with the addition of AgNO_3_, IPA, and EDTA-2Na. However, the degradation rate was significantly inhibited while BQ was added, indicating that ·O_2_^−^ radicals were the main active substances in the degradation process of GTC-300. The ESR is directly used to identify the type of active substance (Figure 9). The yields of ·OH and ·O_2_^−^ in GTC-300 are significantly under illumination. The CB of TiO_2_ is more negative than the potential of O_2_/·O_2_^−^ [44], conducting the reduction of O_2_ to ·O_2_^−^ with generated electrons, and the ·OH can also be partially transformed from ·O_2_^−^ [32]. The above results suggest that GTC-300 promotes the generation of more electrons and facilitates the conversion of O_2_ into ·O_2_^−^.

**Figure 10:**
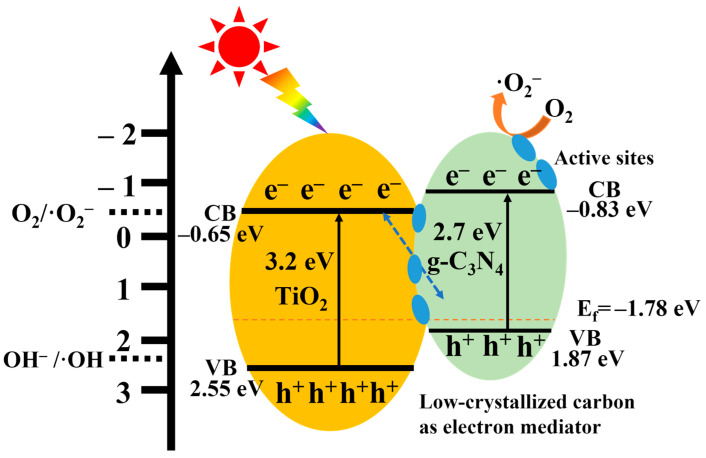
Illustration diagram of the band structure and photocatalytic mechanism of g-C_3_N_4_/C/TiO_2_ nano-heterojunction in situ synthesized by the hydrothermal method.

Based on the above analysis, the energy band structure and a putative mechanism of photocatalytic pollutant degradation for g-C_3_N_4_/C/TiO_2_ are postulated (Figure 10). After investigation, the valence band top of TiO_2_ is 2.55 eV, and the band gap is 3.2 eV, while the valence band top of g-C_3_N_4_ is 1.87 eV, and the band gap is 2.7 eV [45,46,47,48]. When the two form a heterojunction, the Fermi level is flattened. After UPS measurement and calculation, the work function Φ = 6.28 eV was obtained. Further, the Fermi level E_f_ = 1.78 eV (vs. NHE) of GTC-300 was obtained (Figure S8). Under illumination, low-crystallized carbon paired with TiO_2_ and g-C_3_N_4_ can engage in photogenerated electron carrier transition [28]. The weak reductive photoelectrons excited from TiO_2_ could be transferred to carbon very quickly by an electric field driving force. At low-crystallized carbon, photogenerated electrons and holes could then annihilate, extending the distance of charge carrier recombination.

The Z-scheme heterojunction structure g-C_3_N_4_/C/TiO_2_ enhances the photocatalytic response across a wider spectrum, while also enhancing the redox capability and facilitating the migration and separation of photogenerated carriers (Figure 10). The pomegranate-like structure provides a high-quality interface and further improves the carrier transfer efficiency. The presence of low-crystallized carbon not only functions as a solid electron mediator within the Z-scheme heterojunction structure but also serves as a reactive site on the surface, promoting the efficient transport of photogenerated electrons to participate in the degradation reaction, thereby enabling cost-effective and highly efficient pollutant degradation.

## 4. Conclusions

In summary, the pomegranate-like g-C_3_N_4_/C/TiO_2_ nano-heterojunction photocatalyst can be synthesized by the hydrothermal–calcination method. The obtained g-C_3_N_4_/C/TiO_2_ nano-heterojunction photocatalyst has excellent photocatalytic degradation performance on both TC-HCl and MB. The essential low-crystallized carbon has been successfully introduced into the nano-heterojunction, serves as a solid-state electron mediator, and is conducive to carrier migration and photocatalytic capacity. Combining the Z-scheme heterojunction of TiO_2_ with strong redox property and g-C_3_N_4_ with a wide photo-response range, g-C_3_N_4_/C/TiO_2_ utilizes more solar radiation for photocatalytic reaction. In addition, the interface of the pomegranate-like structure with close contact avoiding defects between three components facilitates the separation and transport of photogenerated carriers.

The g-C_3_N_4_/C/TiO_2_ nano-heterojunction can be used in the photocatalytic oxidation process of phenolic compounds and the degradation process of organic compounds or organic solvents. This innovative preparation route has low operational difficulty and low energy consumption, providing ideas for the development of more heterojunction materials.

## Figures and Tables

**Table 1:** Comparison of various literature reports on photocatalytic phenol degradation.

Photocatalyst	TC-HCl (Initial Concentration)	Catalyst Dosage (mg)	Volume (mL)	Degradation (%)	Light Duration (min)	Light Source	Ref.
g-C_3_N_4_/C/TiO_2_	20 mg/L	20	50	87.7%	60	300-W xenon lamp (λ > 400 nm)	This work
g-C_3_N_4_/C/TiO_2_	20 mg/L	20	50	92.9%	40	300-W xenon lamp	This work
TiO_2_@C	20 mg/l	100	100	100%	35	300-W xenon lamp	[34]
CdS-TiO_2_	50 mg/L	50	50	87.06%	480	500-W xenon lamp (λ > 400 nm)	[41]
Ag/TiO_2_/g-C_3_N_4_	60 mg/L	10	50	85.1%	25	300-W xenon lamp	[42]
AgCl/ZnO/g-C_3_N_4_	20 mg/L	60	40	89.05%	50	140 W metal halide lamp (λ > 400 nm)	[43]

## Data Availability

The data will be made available upon request.

## Supplementary Materials

The following supporting information can be downloaded at: https://www.mdpi.com/article/10.3390/nano15050365/s1: Figure S1: TG and DTG pattern of GTC-300 and GTC-500. Figure S2: The nitrogen adsorption-desorption isotherm of (a) GTC-300, (b) GTC-500, (c) TiO_2_/C, and (d) P25. Figure S3: The plots of (ahv)^1/2^ vs. hv of diffuse reflectance spectra for GTC-300, TiO_2_/C, and g-C_3_N_4_ nanoparticles. Figure S4: Enlarged PL spectra for GTC-300, TiO_2_/C, and P25 nanoparticles. Figure S5: UV–Vis absorption curves of GTC-300 for TC-HCl under (a) full-spectrum irradiation and (b) visible light irradiation. Figure S6: (a) Cyclic experiment of GTC-300 and (b) XRD spectra after experiment of GTC-300. Figure S7: UV–Vis absorption curves of GTC-300 (a) and GTC-500 (b) of TC-HCl under full-spectrum irradiation; (c) photocatalytic activity and (d) the rate constant for degradation of MB by GTC-300, P25, TiO_2_/C, and g-C_3_N_4_ under light irradiation. Figure S8: (a) UPS spectrum of GTC-300 and (b) the enlarged spectrum from 2 eV to 6 eV for investigating the work function and Fermi level.
