# Supplementary material for: Low-Crystallized Carbon as an Electron Mediator in g-C3N4/C/TiO2 for Enhancing Photocatalytic Degradation of Antibiotics
Source: Nanomaterials (Basel). 2025 Feb 27;15(5):365. doi: 10.3390/nano15050365 (PMC11901478; doi:10.3390/nano15050365)
Supplement: Supplementary file 1 [file nanomaterials-15-00365-s001.zip › nanomaterials-3481123-supplementary.pdf]

Supplementary Information

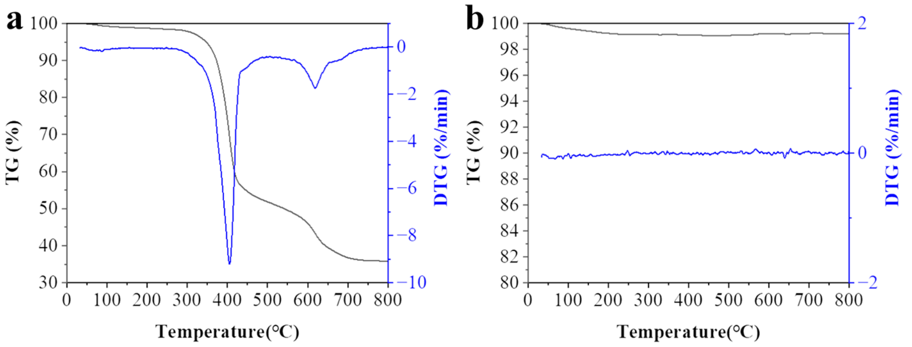

Figure S1 TG and DTG pattern of GTC-300 and GTC-500

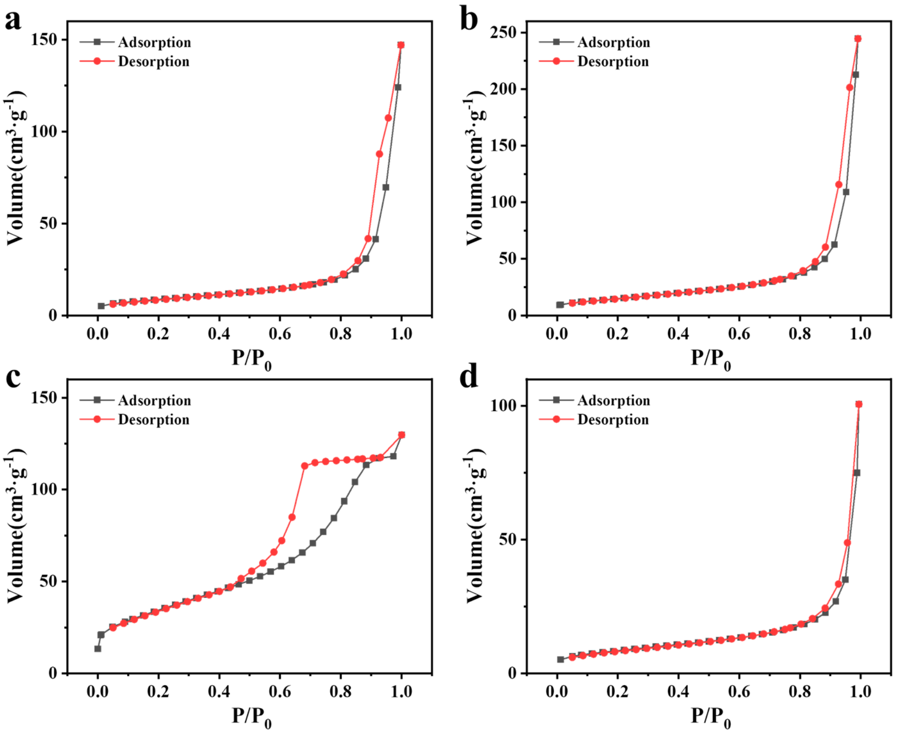

Figure S2 The nitrogen adsorption-desorption isotherm of (a) GTC-300, (b) GTC-500, (c) TiO_2/C and (d) P25

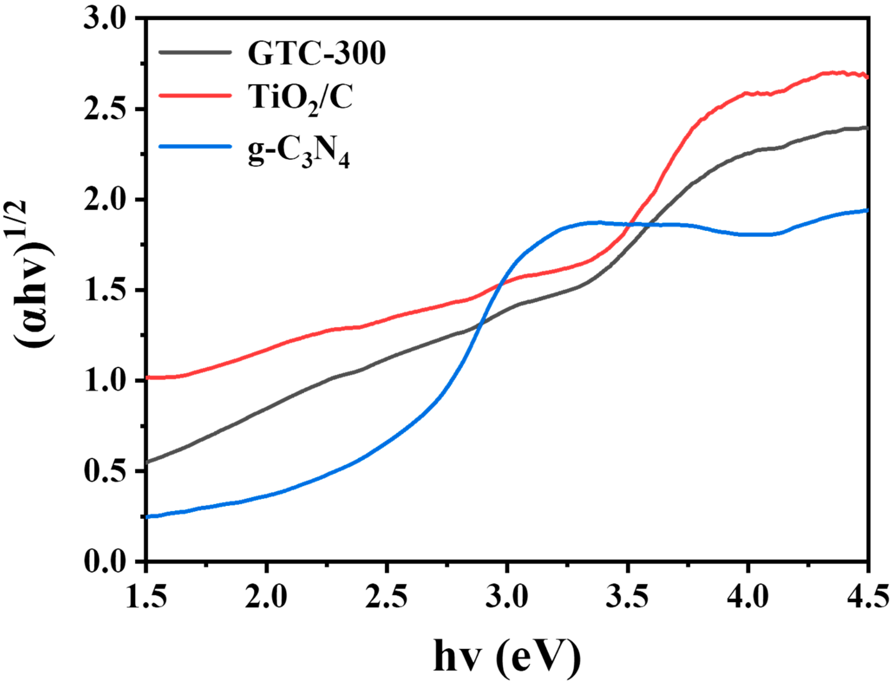

Figure S3 The plots of $(ahv)^{1/2}$ vs. hv of diffuse reflectance spectra for GTC-300, TiO_2/C and g-C_3N_4 nanoparticles

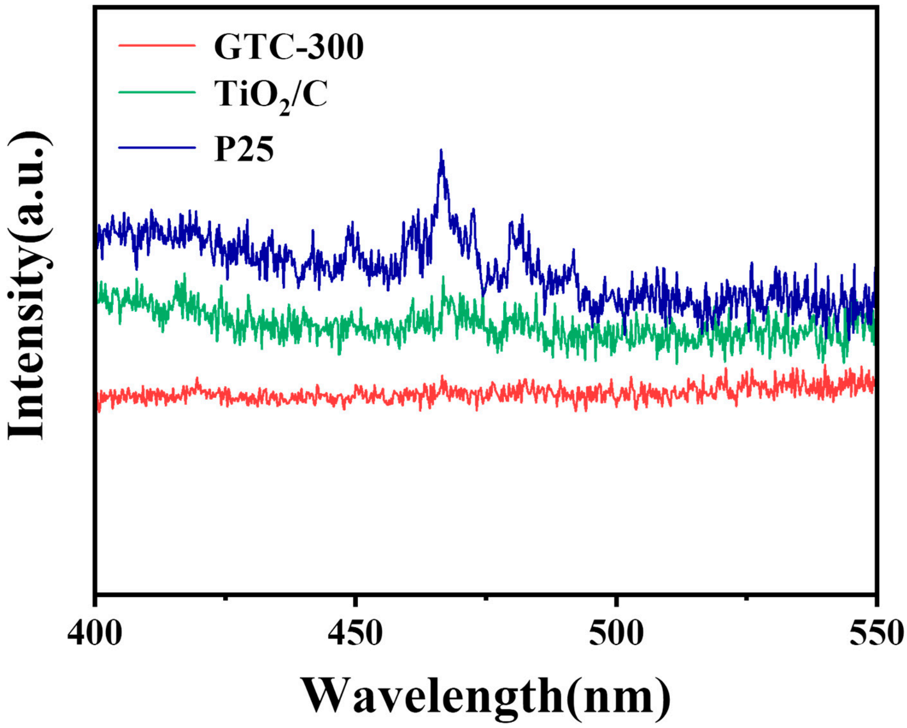

Figure S4 Enlarged PL spectra for GTC-300, TiO_2/C and P25 nanoparticles

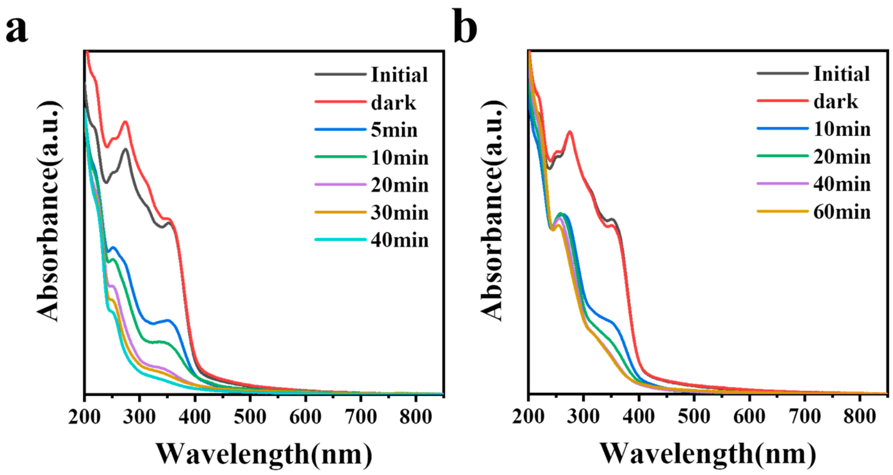

Figure S5 UV–Vis absorption curves of GTC-300 for TC-HCl under (a) full-spectrum irradiation and (b) visible light irradiation

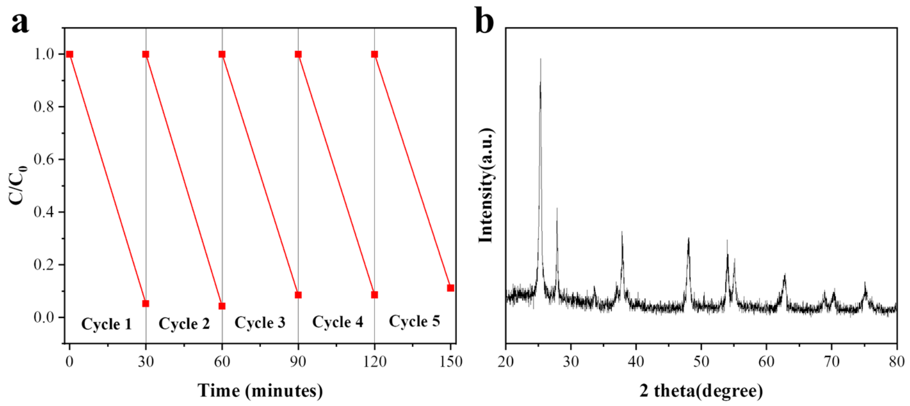

Figure S6 (a) Cyclic experiment of GTC-300 and (b) XRD spectra after experiment of GTC-300

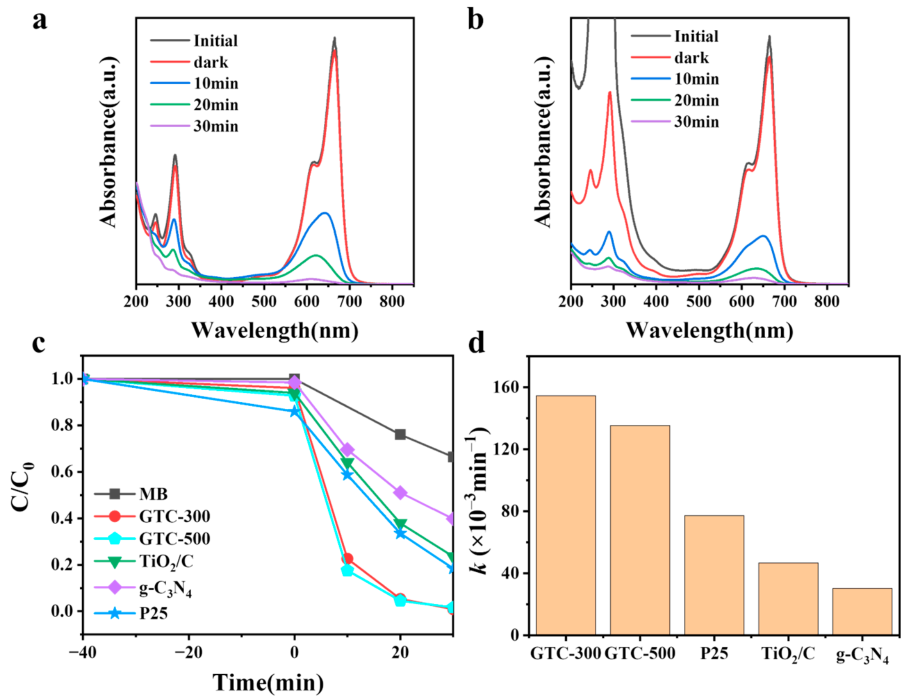

Figure S7 UV–Vis absorption curves of GTC-300 (a) and GTC-500 (b) of TC-HCl under full-spectrum irradiation; (c) photocatalytic activity and (d) the rate constant for degradation of MB by GTC-300, P25, TiO_2/C and g-C_3N_4 under light irradiation

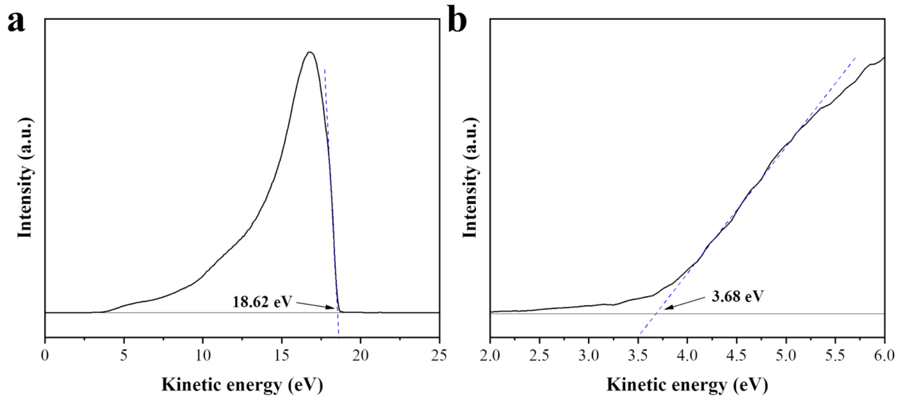

Figure S8 (a) UPS spectrum of GTC-300 and (b) the enlarged spectrum from 2 eV to 6 eV for investigating the work function and Fermi level
